# COVID-19 and Hurricanes: The Impact of Natural Disasters during a Pandemic in Honduras, Central America

**DOI:** 10.1017/S1049023X21000182

**Published:** 2021-02-15

**Authors:** Lysien I. Zambrano, Itzel Carolina Fuentes-Barahona, Karla Iveth Henriquez-Marquez, Walter O. Vasquez-Bonilla, Manuel Sierra, Fausto Muñoz-Lara, Camila Luna, D. Katterine Bonilla-Aldana, Alfonso J. Rodriguez-Morales

**Affiliations:** 1. Department of Physiological and Morphological Sciences, Faculty of Medical Sciences, Universidad Nacional Autónoma de Honduras, Tegucigalpa, Honduras; 2. Latin American Network of Coronavirus Disease 2019 Research (LANCOVID), Pereira, Risaralda, Colombia; 3. Universidad Nacional Autónoma de Honduras, Tegucigalpa, Honduras; 4. Departamento de Ginecología y Obstetricia, Hospital Escuela, Tegucigalpa, Honduras; 5. Departamento de Patología, Hospital General San Juan de Dios, Ciudad de Guatemala; 6. Unidad de Investigación Científica, Facultad de Ciencias Médicas, Universidad Nacional Autónoma de Honduras (UNAH), Tegucigalpa, Honduras; 7. Department of Internal Medicine, Faculty of Medical Sciences, Universidad Nacional Autónoma de Honduras (UNAH), Tegucigalpa, Honduras; 8. Department of Internal Medicine, Hospital Escuela, Tegucigalpa, Honduras; 9. Universidad Cientifica del Sur, Lima, Peru; 10. Semillero de Investigación en Zoonosis (SIZOO), Grupo BIOECOS, Fundacion Universitaria Autonoma de las Americas, Pereira, Risaralda, Colombia; 11. Grupo de Investigación Biomedicina, Faculty of Medicine, Fundacion Universitaria Autonoma de las Americas, Pereira, Risaralda, Colombia

**Keywords:** Central America, COVID-19, hurricanes, natural disasters

Sir,

According to the Global Climate Index, Honduras is classified as one of the nations most vulnerable to natural disasters in the world. It was the scene of the hurricanes in 1974 (Fifi) and 1988 (Mitch),^1^ which have been the most catastrophic weather events that have affected the country, causing human losses, material, economic, and health damages. It took many years and great resources for the reconstruction, which is still incomplete.

**Figure 1.**
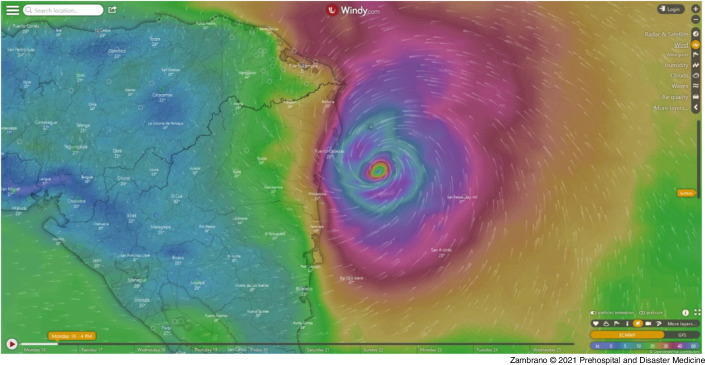
Hurricane Iota Arriving to the Coasts of Central America on November 16, 2020. Source: Open Meteorological Platform Windy (https://www.windy.com/?13.646,-83.046,8).

In November 2020, Honduras became the scene again: two major weather events had occurred in less than two weeks, causing incalculable damage that is still being accounted for (Table 1). The hurricanes Eta and Iota devastated the country (Figure 1), already affected during the last nine months by the COVID-19 pandemic.

In March 2020, the first cases of COVID-19 were reported in Honduras. By November 29, 2020, the national system of health (SINAGER) reported 107,513 confirmed cases and 2,905 deaths.^2^ Besides, the country’s economy has been plunged into a severe crisis by this pandemic and will undoubtedly be aggravated by recent natural disasters. When a decline in the curve of reported COVID-19 cases was finally expected, the new events place the already vulnerable country in an almost impossible situation. At the end of October 2020, the formation of hurricane Eta was announced, a Category 5 hurricane, which would affect the lands of Nicaragua and Honduras. This event caused flood damage in some areas that have the highest reported COVID-19 cases. Some of the victims were refugees in roughly 1,000 shelters with little or no biosecurity measures. Others continue on the streets, along rivers, under bridges, and other public places where the health crisis will worsen. The resurgence of dengue complicates the situation and is endemic in Honduras. Currently, the Health Information Platform for the Americas reported a total of 23,444 cases with a mortality rate of 0.1 per 100,000 population by dengue.^3,4^

**Table 1.** Comparison of Damages During Four Major Hurricanes in Honduras

	Hurricane			
Consequences	Fifi * (1974)	Mitch** (1998)	Eta *** (2020)	Iota **** (2020)
Saffir-Simpson Hurricane Wind Scale	Category 2	Category 5	Category 4	Category 5
Hurricane Scale when Entering Honduras	Tropical Depression	Category 1	Tropical Depression	Tropical Depression
Deceased	Between 5000-8,000	5,657	74	14
Affected People	500,000	1,500,000	3,011,760	664,590
Missing People	ND	8,058	8	1
Evacuated People	130,000	285,000	179,136	184,626
Hostels	ND	1375	1000	ND
Sheltered People	130,000	285,000	86,228	86,570
Recued People	ND	112,272	89,665	ND
Affected or Damaged Homes	12,500	50,000	26,828	19,372
Destroyed Homes	3,000	66,188	60	29
Affected Roads	ND	107	173	87
Damaged Schools	162	2,800 rooms of schools in primary education	8	2
Damaged Bridges	120	More than 200	43	19
Destroyed Bridges	18	189	32	13
Emergency Aid to Honduras	USD 2,271,980	USD 38,000,000	USD 9,000,000	ND
Post-Climate Event Diseases	Dysentery Typhoid Malaria	Gastrointestinal infections: Cholera (3 cases) Leptospirosis (172 cases)	ND	ND

Data Sources:

* https://repositorio.cepal.org/bitstream/handle/11362/15031/S7400458_es.pdf?sequence=1&isAllowed=y (Access December 1, 2020); http://cidbimena.desastres.hn/ri-hn2/pdf/doch0014/doch0014.htm (Accessed December 1, 2020).

** http://cidbimena.desastres.hn/ri-hn/pdf/spa/doc12140/doc12140-contenido.pdf (Accessed November 30, 2020); http://cidbimena.desastres.hn/ri-hn/pdf/spa/doc12921/doc12921-a.pdf (Accessed November 20, 2020).

*** https://reliefweb.int/sites/reliefweb.int/files/resources/Honduras%20-%20Huracanes%20Eta%20e%20Iota%2020Reporte%20de%20situaci%C3%B3n%20No.%2020%20%2822-11-2020%29%201700%20h.pdf (Accessed November 30, 2020); https://reliefweb.int/sites/reliefweb.int/files/resources/SitRep%204%20Tormentas%20ETA%20IOTA%20Honduras%202020.pdf (Accessed November 30, 2020).

**** http://sigmof.icf.gob.hn/?page_id=7546 (Accessed November 20, 2020); https://www.oncenoticias.hn/galerias-el-paso-arrasador-de-los-huracanes-mitch-y-fifi-en-honduras/?__cf_chl_jschl_tk__=dc82a5fd7b0cb0ee6cd1527ccfb00a71041da1db-1606673591-0 XyaHF9vxi_rPHR1BXLT1EAV8EMhs_Aix0W6i59TjJPPaLeALhzrBaOt4w09gzIgiC1HjyeriPMYxY2GAs_G92SCZ1gM6tq3yqthwarHMo2hzXRasg0D1EF3rVAfa0c1mGB41Nl8dh3aC3UmQA5xamnn6V5HdIWiS-QsIjAwYimMTJY8pVUZWj9ANGPd05yxhQmCg7q45AOkzodP2JsUqmuC_uuEtUhbNal8XGKVtG4r4nro39OVYYgkBvMEC8EFW9jQ7AgDGr0mXxbIqA44YD_-7u2VYNnv8xKM8m0M-pM1SkhEHyirwiPqEFMlYYwwjL9qZUhZcANLPKoDRnnJBDO33n1nJo6AEg_Lxjssv67XJ5gdlpQnvaBSW51v5b2_EwrbAk46B4bJ2qyT9TA_3xM (Accessed November 20, 2020); Mitch: http://cidbimena.desastres.hn/ri-hn/pdf/spa/doc11026/doc11026-1.pdf (Accessed December 1, 2020); https://www.bbc.com/mundo/noticias-america-latina-54965248 (Accessed December 1, 2020).

ND: No Data Available.

It is expected that the emerging diseases that usually appear after these climatic phenomena will appear (arboviruses, leptospirosis, malaria, cholera, a considerable increase in dermatological diseases, and an increase in psychological disorders). Not to mention all the acute and chronic pathology that usually occur in the country, which is managed inefficiently.^5^

Although the floods caused by the overflowing of the rivers have caused most of the damages, the supersaturation of the soils with possible landslides will increase material losses.^6^ The reports of the Secretary of Agriculture about losses or affectations in around 374,000 acres in essential grain crops, such as banana, sugar cane, citrus, cocoa, rambutan, among others, are concerning.^7^ Also, human losses and deterioration in living conditions in the affected population are reaching threatening levels.

To this day, it is difficult to measure the current and future consequences on the socio-economic development of the country, in this uncertain scenario the increase in health demand in an already collapsed health system in the face of the summation panorama: COVID-19, emerging infections, endemic diseases, common diseases, plus two major natural disasters, turn this situation into a humanitarian crisis without precedent in the history of Honduras.

The aftermath of the floods and the probable rebound in COVID-19 cases should alert us not to give up and increase biosecurity measures as much as possible and provide social assistance to more than one million people affected and thousands more at risk in Honduras and other countries of Central America.^8,9^ The challenges for the future are innumerable and of great magnitude, the leadership of the different experts in each area is required to be able to solve in a logical, harmonious, fair, and efficient way the situation on all fronts, probably being: health, education, economy, infrastructure, and the severe problem of insecurity being the main ones. It is not an easy task that must be accomplished quickly; otherwise, the consequences for the country are unpredictable.
